# A Novel Pre-Clinical Murine Model to Study the Life Cycle and Progression of Cervical and Anal Papillomavirus Infections

**DOI:** 10.1371/journal.pone.0120128

**Published:** 2015-03-24

**Authors:** Nancy M. Cladel, Lynn R. Budgeon, Karla K. Balogh, Timothy K. Cooper, Jiafen Hu, Neil D. Christensen

**Affiliations:** 1 The Jake Gittlen Laboratories for Cancer Research, Pennsylvania State University College of Medicine, Hershey, PA, United States of America; 2 Department of Pathology, Pennsylvania State University College of Medicine, Hershey, PA, United States of America; 3 Department of Microbiology and Immunology, Pennsylvania State University College of Medicine, Hershey, PA, United States of America; 4 Department of Comparative Medicine, Pennsylvania State University College of Medicine Hershey, PA, United States of America; National Institute of Health - National Cancer Institute, UNITED STATES

## Abstract

**Background:**

Papillomavirus disease and associated cancers remain a significant health burden in much of the world. The current protective vaccines, Gardasil and Cervarix, are expensive and not readily available to the underprivileged. In addition, the vaccines have not gained wide acceptance in the United States nor do they provide therapeutic value. Papillomaviruses are strictly species specific and thus human viruses cannot be studied in an animal host. An appropriate model for mucosal disease has long been sought. We chose to investigate whether the newly discovered mouse papillomavirus, MmuPV1, could infect mucosal tissues in *Foxn1^nu^/Foxn1^nu^* mice.

**Methods:**

The vaginal and anal canals of *Foxn1^nu^/Foxn1^nu^* mice were gently abraded using Nonoxynol-9 and “Doctor’s BrushPicks” and MmuPV1 was delivered into the vaginal tract or the anal canal.

**Results:**

Productive vaginal, cervical and anal infections developed in all mice. Vaginal/cervical infections could be monitored by vaginal lavage. Dysplasias were evident in all animals.

**Conclusions:**

Anogenital tissues of a common laboratory mouse can be infected with a papillomavirus unique to that animal. This observation will pave the way for fundamental virological and immunological studies that have been challenging to carry out heretofore due to lack of a suitable model system.

## Introduction

Despite the introduction of two protective vaccines, Gardasil and Cervarix, in the past decade, papillomavirus disease remains a major global health challenge. Cervical cancer, which is almost always associated with high risk human papillomaviruses (HPVs), is a leading cause of death of women of childbearing age in the developing world [1]. Head and neck and anal cancers are also associated with HPV [2]. Cases of anal cancer are steadily rising as the population of immunocompromised individuals increases [3]. The current protective vaccines are expensive and require a series of two (9–13 year old children) or three immunizations insuring that their use is not practical for many of the world’s most vulnerable citizens. In addition, the vaccines protect against only a subset of the human papillomavirus types associated with malignancies. There is no effective therapeutic vaccine and the efficacy of other therapeutic interventions is limited. For many decades, the research community has sought a small animal model to facilitate the study of anogenital disease and cancers associated with papillomaviruses. In 2011, Ingle et al reported the discovery of a new mouse papillomavirus, MmuPV1, the first papillomavirus to infect a common laboratory mouse [4]. The virus was reported to be restricted to cutaneous tissues. A subsequent report by Handisurya *et al* supported this finding [5]. Our first studies indicated that the virus also had mucosal tropism [6]. In this paper we describe work that confirms the dual tropism of the virus and paves the way for the first practical small animal model to allow for the study of papillomavirus infections in anogenital tissues. Among the topics amenable to study are those of tissue tropism, latency, the role of the estrus cycle in disease, transmission (both vertical and horizontal) and, potentially, cancers of the anogenital tract. MmuPV1 is a member of the pi papillomavirus family and thus evolutionarily distinct from the alpha viruses most often associated with human cancers. Nonetheless it shares important features with the alpha viruses including the presence of oncogenes E6 and E7, a non-coding region with many of the same regulatory and transcription factor binding sites [7] and a tropism that includes the anogenital tract. This model meets the requirements established by the papillomavirus community and will be of considerable use to investigators in the field.

## Materials and Methods

### Viral stock

Virus was isolated from lesions on the tails of mice from our previous study [6]. The lesions were scraped from the tail with a scalpel blade and homogenized in phosphate buffered saline (PBS) using a Polytron homogenizer (Brinkman PT10–35) at highest speed for three minutes while chilling in an ice bath. The homogenate was spun at 10,000 rpm and the supernatant was decanted into Eppendorf tubes for storage at -20°C. For these experiments, the virus was diluted 1:5 in PBS and 200μl was passed through a 0.2μm cellulose acetate sterile syringe filter. This was chased by the addition of 200μl PBS. The PBS filtrate was added to the filtered virus to give a total of 250μl sterile virus solution when taking into account loss in the filter. Viral DNA was quantitated by extraction of the DNA from 5μl of this stock. The DNA was isolated in 50 μl water and a SYBR green Q PCR assay was run on a series of 1:2 dilutions. Dilutions of plasmid DNA of known concentration were used for the standard curve. One μl of DNA was determined to contain 0.012ng viral DNA. 1 ng contains 1.2 x10^8^ copies of viral DNA (http://cels.uri.edu/gsc/cndna.html). Thus, 1 μl of the DNA extract contains 1.4x10^6^ copies of viral DNA meaning that 1 μl of the viral extract contains 1.4 x 10^7^ viral genome equivalents.

### Vaginal infections

All mouse work was approved by the Institutional Animal Care and Use Committee of Pennsylvania State University’s College of Medicine. Outbred Hsd Foxn1nu/*Foxn1* ^*nu*^ mice were obtained from Harlan Laboratories and were housed in sterile cages within sterile filter hoods and were fed sterilized food and water. At age 13 weeks, mice were inoculated subcutaneously with 3mg progesterone in the form of Depo-provera (Pfizer) in 100microliters PBS. Progesterone has been shown to facilitate infection of sexually transmitted disease in mouse models [8]. Three days following injection, the mice were sedated i.p. with 0.1ml/10g body weight of ketamine/xylazine mixture (100mg/10mg in 10mls PBS). Doctors’ Brush Picks (available in the dental section of most pharmacies) were coated with Conceptrol (Ortho, available over the counter), inserted into the vaginal canal and rotated gently 15 times to create minor abrasions. Conceptrol contains 4% Nonoxynol-9 (N-9), which has been shown to be essential for pseudovirus infection in the vaginal canal of mice [9]. Twenty-four hours later, the mice were again anaesthetized. Into the vaginal canals of the animals was pipetted 25μl of the sterilized viral suspension using a 200μl light touch pipette. The Brush Pick was used to gently abrade the site and to facilitate penetration of the virus. A second aliquot of 25 μl of virus was added and the abrasion was repeated. Total virus delivered was calculated to be 6 x 10^8^ viral particles per mouse. Animals were placed on their backs during recovery to minimize loss of virus from the vaginal canal. Monitoring was conducted weekly and a photographic log was created for each animal.

### Anal infections

*Foxn1* ^*nu*^/*Foxn1* ^*nu*^ mice were maintained as for the vaginally infected animals. At age 12 weeks, mice were anaesthetized as for the vaginally infected animals. The anal canals were gently abraded with Doctor’s Brush Picks by rotating back and forth 20 times. Both the exterior and the interior of the canal were abraded in this manner. The Brush Pick was then covered with Conceptrol and the Conceptrol was delivered to the anus. Mice were left for 24 hours. They were again anaesthetized and into the anus of each was pipetted 10μl (1.4x10^8^ viral particles) of sterile viral stock. Monitoring was conducted weekly and a photographic log was created for each animal.

### Vaginal smears

Vaginal lavage was conducted using 40μl sterile PBS introduced into the vaginal canal with an adjustable pipette and a disposable tip. The PBS was gently pipetted in and out of the vaginal canal before spreading onto glass microscope slides. The slides were air-dried and fixed in 10% neutral buffered formalin (NBF). Standard H and E and GSA staining were performed as noted below.

### Harvest and analysis of tissues

Following euthanasia, a central subcutaneous abdominal incision was made and the large and small intestines were removed. The remainder of the animal was placed in NBF for 24 hours before the anorectal and reproductive organs were carefully excised *in toto*. The specimen was routinely processed to paraffin blocks. Sequential sections were cut for H and E analysis, *in situ* hybridization and immunohistochemistry. Standard conditions were used for H and E staining. A subgenomic fragment of MmuPV1 (3913bp EcoRV/BamH1 fragment) was used as an *in situ* hybridization probe for the detection of MmuPV1 DNA in tissues. The probe was biotinylated using the random priming method and diluted in a hybridization cocktail described in previous work [10]. Access to target DNA was obtained with 0.2 mg/ml pepsin in 0.1N HCl incubation at 37°C for 8 min. After thorough washing, the biotinylated probe was applied and heated to 95°C for 5 min. to achieve dissociation of target and probe DNA. Reannealing was allowed to occur for 2 hours at 37°C. Target- bound biotin was detected using a streptavidin AP conjugate followed by colorimetric development in BCIP/NBT. ISH was performed similarly for lavage smears. Notable modifications were the reduction of pepsin concentration to 0.1mg/ml and DNA dissociation time to 3 minutes to accommodate the fragility of monolayer cell adhesion. For immunohistochemical (IHC) L1 capsid detection, a goat Group Specific Antibody (GSA) to a conserved region of L1 (ViroStat #5001) was used on FFPE sections. Detection was achieved using the ImmPRESS anti-goat IgG polymer system (Vector #MP-7405).

## Results

### The vaginal canal and cervix of *Foxn1* ^*nu*^/*Foxn1* ^*nu*^ mice are susceptible to infection with MmuPV1

**Fig 1:**
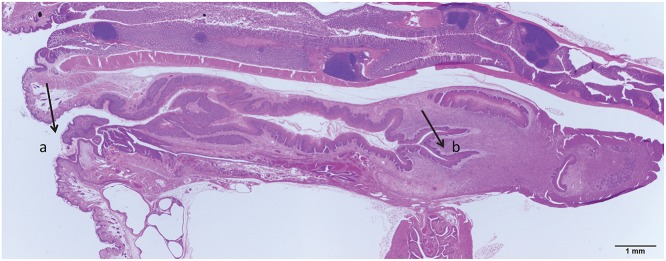
Representative whole mount of the mouse anogenital tract. In this section the introitus (a) and cervix (b) as well as the entire vaginal tract are clearly seen.

**Fig 2:**
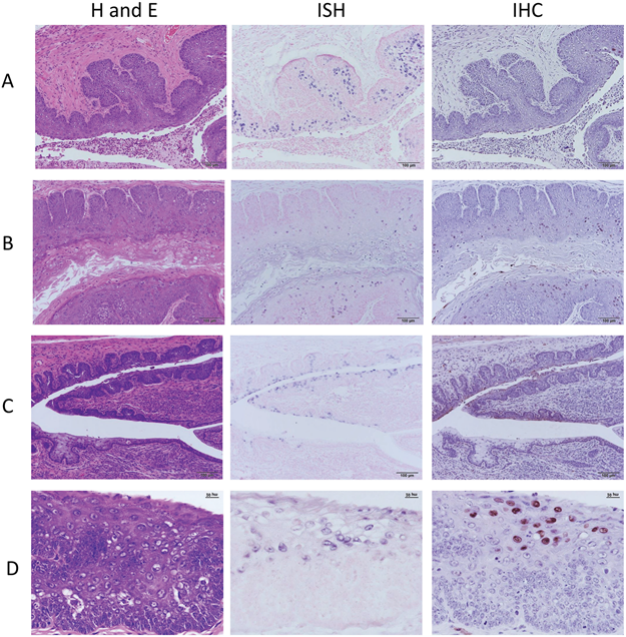
A-D H and E, *in situ* hybridization and immunohistochemistry at four time points. A) Time point 9 weeks post infection. Vaginal lesions; ISH positive cells greatly outnumber IHC positive cells. 20X B) Time point 14 weeks post infection. Vaginal lesions exhibit greatly increased IHC signal. 20X C) Time point 25 weeks post infection. Endocervical canal and dorsal fornix. ISH positive squamous and mucous cells are present in the caudal canal. Only squamous cells are IHC positive. 20X D) Time point 30 weeks post infection. Cytologic atypia in the stratified squamous vaginal epithelium, 40X.

Vaginally infected mice were sacrificed at weeks 9, 14, 25, and 30 post infection. A representative sample of the whole mount of the anogenital region is shown in Fig. 1. Fig. 2A-D is illustrative of the findings in the vaginally infected animals. Fig. 2A represents the histology of a mouse sacrificed at nine weeks post infection. By this time point, the animal had developed MmuPV1 infection involving a substantial portion of the vaginal epithelium as determined by strong *in situ* hybridization signal. Characteristic of the infected tissue were cells showing 1–2 prominent nucleoli, intranuclear cytoplasmic invaginations, and nuclear molding. Capsid antigen positivity, although present, was not abundant. Pathology of the lesions was analogous to VAIN1 (vaginal intraepithelialneoplasia1) with some lesions approaching VAIN2 (S1 Fig. A). Dysplastic squamous epithelium was observed to be undermining adjacent cervical glandular epithelium. This is reminiscent of squamous metaplasia of the cervical glands in primates. (S1 Fig. B). Fig. 2B shows the histology of an animal sacrificed at 14 weeks post infection. Abundant infected cells were present throughout the length of the vaginal canal. Infected cells were seen within the *stratum spinosum* and *corneum* and had amphophilic cytoplasm, large vesicular nuclei and occasional nuclear molding. Dysplasia was analogous to VAIN1. IHC positivity for capsid antigen was more extensive than in the mouse sacrificed at week nine post infection. Cervical infection was present in both the ectocervix and the caudal endocervical canal where both ISH and IHC signals were abundant (S2 Fig. A-C).

Endocervical infection is illustrated in Fig. 2C. This mouse was sacrificed at week 25 post infection. Both squamous and mucous cells were ISH positive but only squamous cells were IHC positive. Vaginal epithelium was strongly positive both by ISH and IHC. S3 Fig. is illustrative.

Fig. 2D represents histology found at the final time point of the study, week 30 post infection. Viral DNA was detected throughout the vaginal canal and ectocervix by *in situ* hybridization and capsid antigen signal was extensive. Dysplasia was seen throughout.

### Both cutaneous and mucosal anal tissues of the *Foxn1* ^*nu*^/*Foxn1* ^*nu*^ mouse are susceptible to MmuPV1 infection

**Fig 3:**
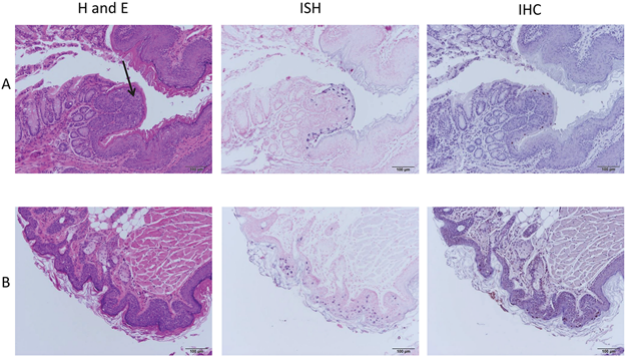
Anal infection, 16 weeks post infection. A) There is strong ISH positivity in cells near the anorectal junction (arrow) as well as strong IHC positivity. 10X. B) There is mild acanthosis and dysplasia in the haired (cutaneous) skin just dorsal to the anus. Both ISH and IHC are positive. 10X.

Mice infected in the anal canal were sacrificed at weeks 16 and 24. Sequential sections of the whole mount were examined by H and E for histology, in situ hybridization for MmuPV1 DNA positivity, and by immunohistochemistry for capsid antigen. Histology of anally infected animals is shown in Fig. 3A-B. The mouse represented in these figures was sacrificed at 16 weeks post infection. Fig. 3A shows focal low-grade dysplasia with frequent amphophilic cells in the mucosal tissue near the rectoanal junction, the site of most malignant transformation. The cells are strongly positive for viral DNA and for capsid antigen. Fig. 3B shows positive signal, mild acanthosis, and dysplasia in the haired (cutaneous) skin just dorsal to the anus. Infection in this mouse thus demonstrates the dual tropism of MmuPV1.

### Vaginal/cervical infections can be monitored by vaginal lavage

**Fig 4:**
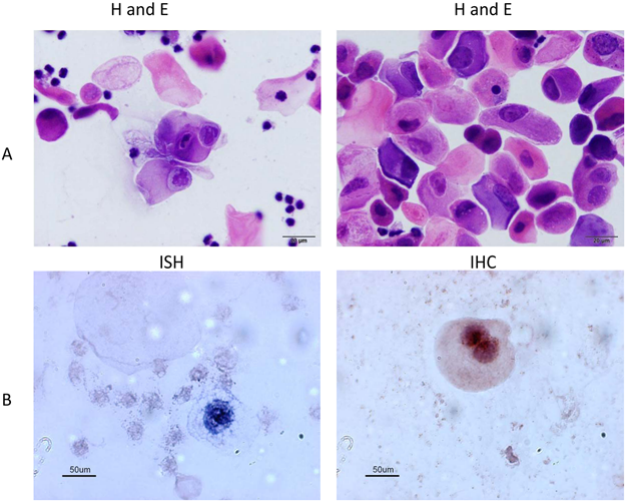
Vaginal smear 24 weeks post infection. A) Cytology. Highly cellular sample consisting of very large numbers of fully cornified anucleate squames and nucleated squamous cells. Frequent atypical squamous cells with ribbon- like central chromatin and abundant amphophilic cytoplasm (resembling inclusion). B) ISH and IHC are both positive for MmuPV1.

In order to determine if vaginal smears could be used to follow the course of infection, vaginal lavage was done on vaginally infected animals at 24 weeks post infection. Fig. 4A shows H and E staining showing fully cornified anucleate squames and a number of atypical squamous cells, many with ribbon-like central chromatin and abundant amphophilic cytoplasm resembling inclusion seen with viral particles by histology. Fig. 4B shows *in situ* hybridization and immunohistochemistry performed on the smears. Both demonstrated the presence of virus. PCR amplification on DNA extracted from a portion of the lavage confirmed the presence of abundant viral DNA (data not shown). The results demonstrated that, as for the human, vaginal smears provide a minimally invasive tool to monitor papillomavirus infections in the mouse vaginal tract.

## Discussion

When MmuPV1 was first described in 2011, it was reported to be a cutaneous papillomavirus[4]. Our laboratory has used the cutaneous Cottontail Rabbit Papillomavirus (CRPV) and the mucosal Rabbit Oral Papillomavirus (ROPV) for many years to study the basic biology of the virus as well as immunological aspects of infection [11–22] and they have proved to be excellent pre-clinical models. Other laboratories have also used the CRPV model to good advantage [23–37]. However reagents for the rabbit are somewhat limited and housing costs are substantial. In addition, the ROPV model has presented some technical challenges that have presented unanticipated roadblocks (unpublished data). We were interested to determine whether we could establish the MmuPV1/mouse model as an adjunct to our rabbit models. In our first study, reported in 2013 (6), we demonstrated the production of the MmuPV1 virus from synthesized viral DNA and the successful infection of cutaneous sites such as the tail. In that study we observed that secondary infections developed over time and that some of the targeted sites were mucosal epithelium. We followed up on that observation and in this paper we report the successful direct infection of vaginal, cervical and anal tissues with MmuPV1 in *the Foxn1* ^*nu*^/*Foxn1* ^*nu*^ mouse.

The papillomavirus research community has long sought a small animal model system to study anogenital papillomavirus disease. With the demonstration that anogenital tissues of a common laboratory mouse can be infected with a virus specific to that animal, we have paved the way for this long sought model. The model can be envisioned to be used in many ways. One of the most important needs is a system to study cervical dysplasias and cancers in an *in vivo* model. While frank cancers were not noted in the short term of the experiments described here, dysplasias were observed, some akin to VAIN2. In our previous study [38], pre-malignant lesions were also noted. It is therefore reasonable to expect that this model may be developed to study papillomavirus-associated cancers of the anogenital tract. Genomes with codon-modifications in the oncogenes may be of use in accelerating malignant progression, as reported for our studies with the CRPV/rabbit model [39].

An extensive repertoire of reagents is available for the laboratory mouse and numerous strains of transgenic mice have been developed. Techniques for the reconstitution and/or depletion of components of the mouse immune system are also well-established. These tools and reagents will be useful in helping to determine those parts of the complex immune system that are crucial to papillomavirus infection and eradication. This work has been initiated for cutaneous MmuPV1 lesions by Handisurya *et al* in a number of immunocompetent mouse strains[40]. The model will also provide unique opportunities to study male to female, female to male, anal to vaginal, vaginal to anal and mother to child transmission and to investigate tissue tropism as it relates to papillomavirus infections. The model may also become a useful tool to study latent disease. The latter topic is of considerable interest to the research and clinical communities in an era when increased numbers of individuals with compromised immune systems are experiencing significant exacerbations of papillomavirus infections [41]. These possibilities and others make this new model a major step forward in efforts to understand the etiology of papillomavirus disease, malignant progression, response to immunity, and disease eradication in mucosal tissues.

Animal models that have been and/or are in use for the study of papillomaviruses are listed in Table 1 along with their strengths and deficiencies. Not a single model is practical for the study of anogenital infections in its natural host. For example, the vaginal mouse model reported in 2007[9] and the simian model published in 2011[42], while presented as papillomavirus infection models, are pseudovirus delivery systems that deliver non-papillomavirus plasmids. They have value in the study of aspects of viral entry but deliver transiently expressed reporter genes in bacterial plasmids, and cannot be considered virus infection models. The CRPV model [43,44], which our laboratory has used extensively, is a cutaneous model and cannot be used to study infections in the sites of greatest medical significance, the vaginal tract, the anus and the oral cavity. The athymic mouse model, reported here, is well suited to fill this void and will be of significant interest to the papillomavirus research community.

**Table 1:** Animal models that have been or are currently being used for papillomavirus research; strengths and deficiencies are noted.

Model	Strengths	Deficiencies
Bovine[46]	Abundant production of virus. Different strains of virus, both mucosal and cutaneous.	Animals are too large to be practical for use in laboratory studies.
Primate[47]	Closely allied with the human system.	High cost and challenging for many institutions.
Canine[48]	Both mucosal and cutaneous.	Lesions regress rapidly. High cost.
Rabbit CRPV model[43,44]	NZW rabbit is a docile laboratory animal. Cancers occur without applied cofactors. Both progressive and regressive strains are available. DNA is infectious.	Virus is cutaneous-tropic. Minimal virus is produced in the domestic rabbit. Wild rabbits, the natural host, are difficult to house and maintain.
Rabbit ROPV model [49]	Mucosal virus infects both tongue and genital tissues. Natural host is the domestic rabbit. Lesions are highly productive of virus.	Lesions regress quickly. Virus is somewhat unstable and viral DNA is difficult to clone in bacteria.(unpublished observations)
Transgenic mouse models[3,50,51]	Oncogenes E6 and E7 can be studied both separately and together.	The virus, itself, is not present in these animals. Oncogenes are expressed from heterologous promoters.
Mastomys coucha model[52]	Genital tissues are among those infected. Animals are co-infected with McPV2 and MnPV allowing for study of dual infections.	Host is a wild rodent from Africa. There is a single outbred colony in the laboratory in Germany.
Pseudovirus infections in mouse and simian vaginal tissues[9,42]	Early entry events can be studied using reporter gene encapsidated by human papillomavirus capsids.	NOT a virus infection, although often described as such. Reporter protein signal is transient and does not persist beyond 72 hours.

### Notes

#### Recent new data to support this work

Since the initial submission of this paper, Sundberg et al [45] published a paper on MmuPV1 that substantiates our findings with respect to vaginal infections and reports malignant potential, albeit at cutaneous sites. These findings strengthen the MmuPV1 model and lend support to our hypothesis that the model will be very useful to plumb many of the unanswered questions relating to papillomavirus infection and consequences.

## Supporting Information

S1 Fig Vaginal infection nine weeks post infection . A: Vaginal changes approach VIN II; frequent mitoses are seen. B: Dysplastic squamous epithelium of the caudal cervical canal is undermining adjacent cervical glandular epithelium. Left 10X; right 40X.
(TIFF)

S2 Fig Vaginal infection fourteen weeks post infection . A: (10X) and B: (40X). H and E shows dysplasia in the caudal vaginal wall and ISH and IHC show abundant signal in the same dysplastic areas. C: H and E (10X), ISH and IHC (40X). The ectocervix stains strongly for both viral DNA and capsid antigen.
(TIFF)

S3 Fig Vaginal infection 25 weeks post infection. Caudoventral vagina shows dysplasia and strong ISH and IHC signals. Koliocytes are present and there is abundant amphophilic cytoplasm. 40X.
(TIFF)
