# Supplementary figures and images for: A Novel Pre-Clinical Murine Model to Study the Life Cycle and Progression of Cervical and Anal Papillomavirus Infections
Source: PLoS One. 2015 Mar 24;10(3):e0120128. doi: 10.1371/journal.pone.0120128 (PMC4372414; doi:10.1371/journal.pone.0120128)

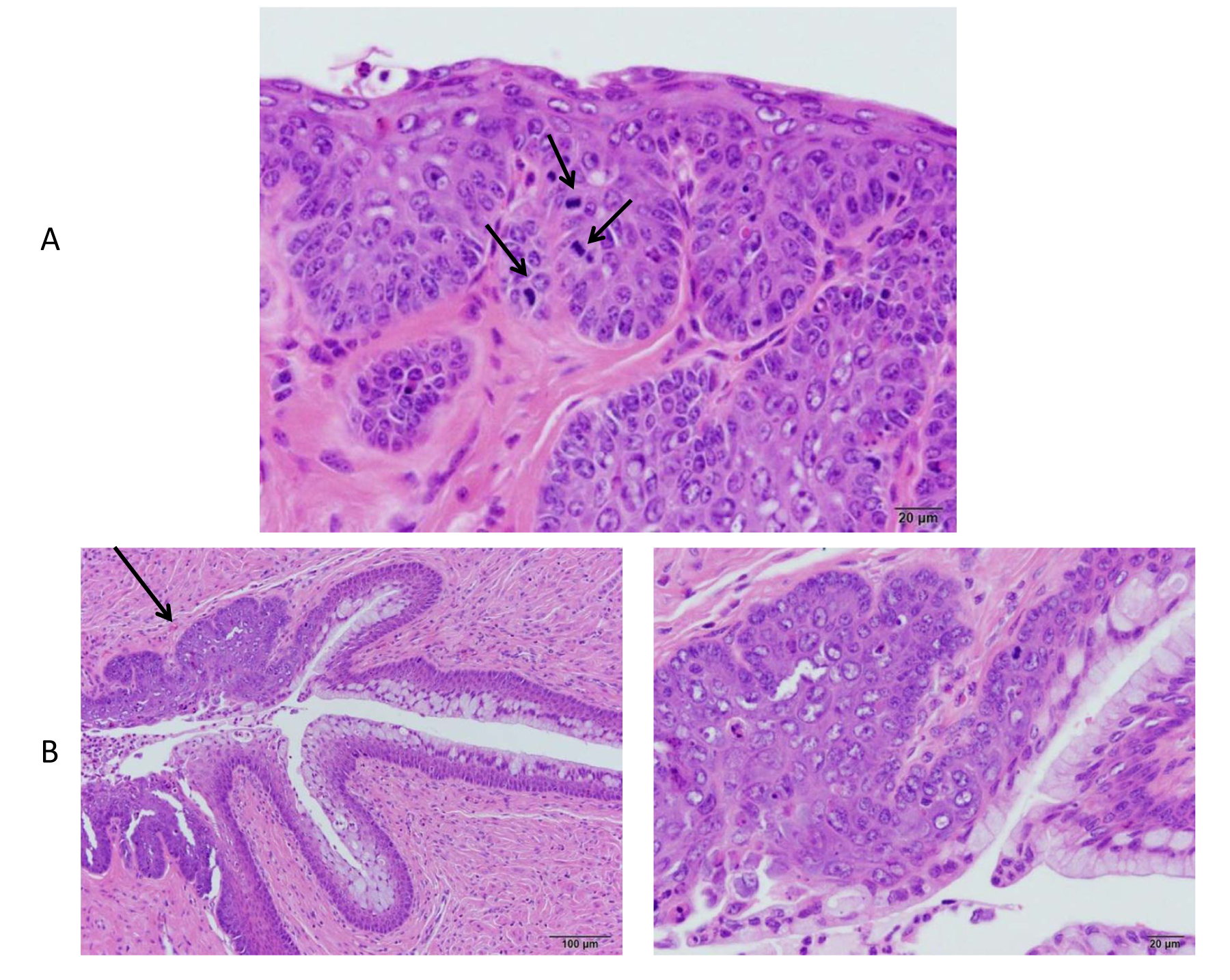

Supplement: S1 Fig — . A: Vaginal changes approach VIN II; frequent mitoses are seen. B: Dysplastic squamous epithelium of the caudal cervical canal is undermining adjacent cervical glandular epithelium. Left 10X; right 40X. (TIFF) [file pone.0120128.s001.TIFF]

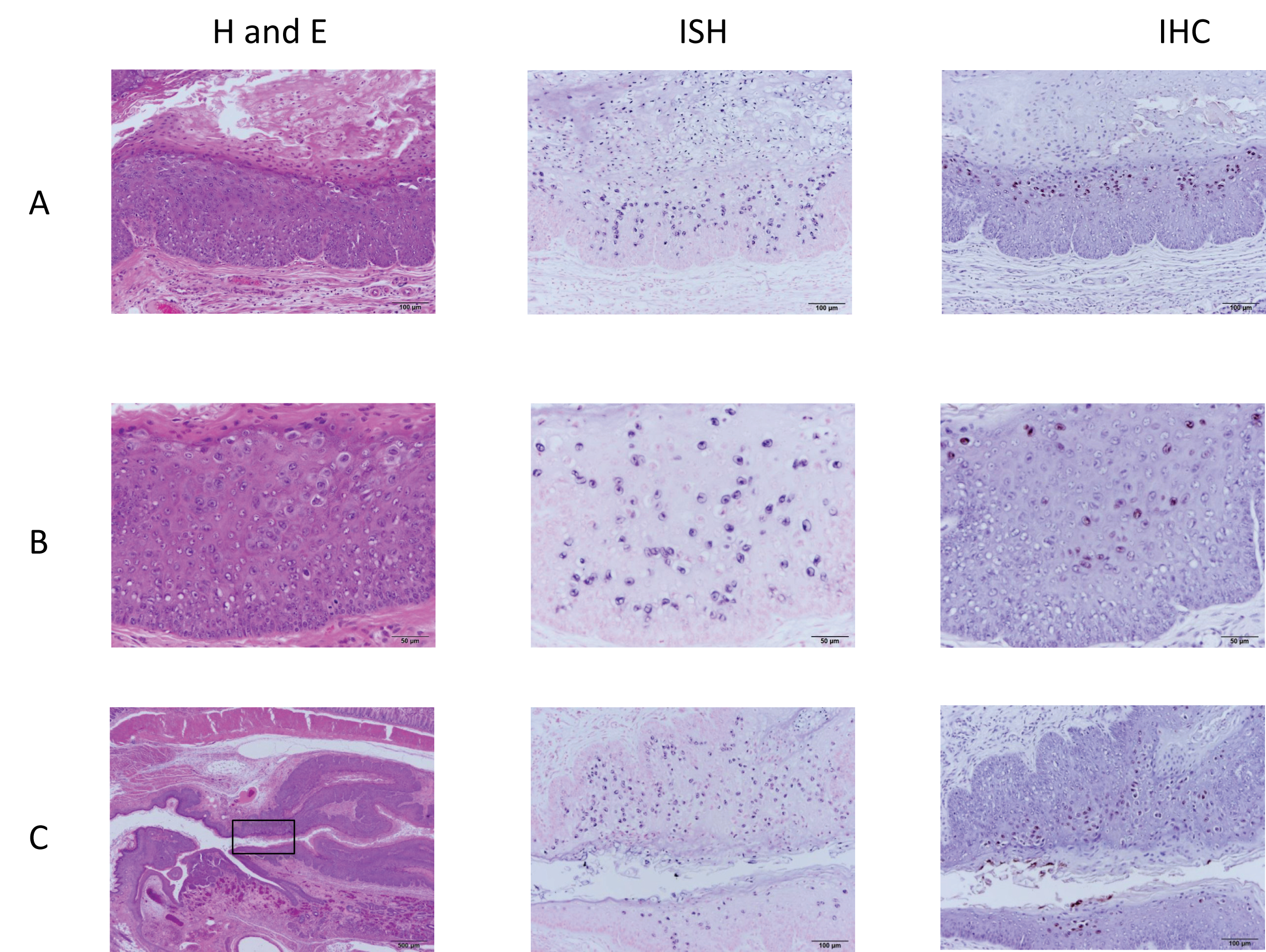

Supplement: S2 Fig — . A: (10X) and B: (40X). H and E shows dysplasia in the caudal vaginal wall and ISH and IHC show abundant signal in the same dysplastic areas. C: H and E (10X), ISH and IHC (40X). The ectocervix stains strongly for both viral DNA and capsid antigen. (TIFF) [file pone.0120128.s002.TIFF]

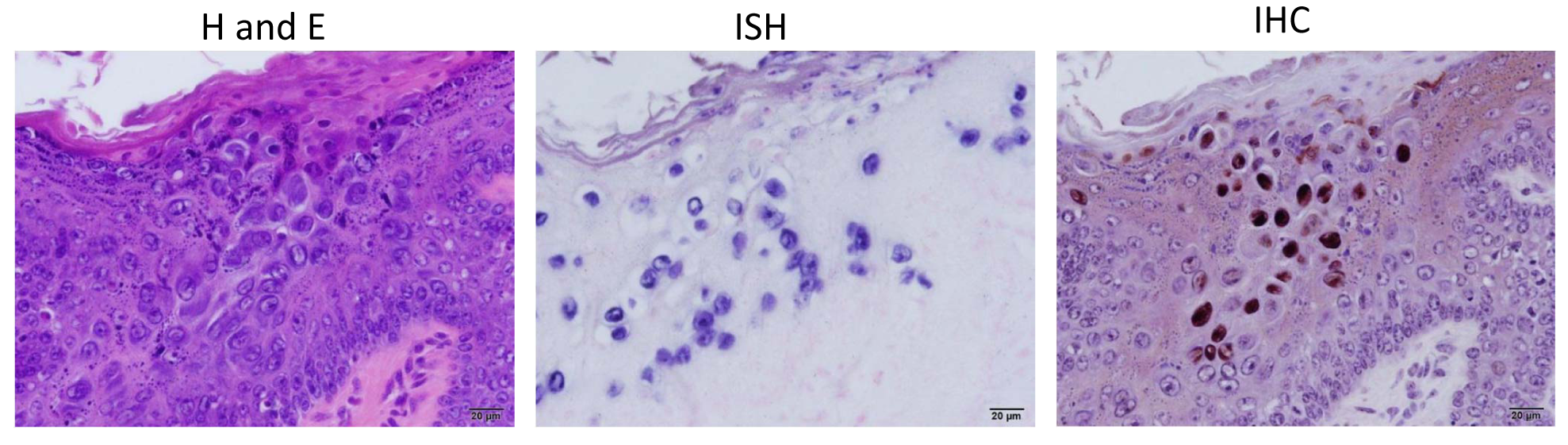

Supplement: S3 Fig — Caudoventral vagina shows dysplasia and strong ISH and IHC signals. Koliocytes are present and there is abundant amphophilic cytoplasm. 40X. (TIFF) [file pone.0120128.s003.TIFF]
